# The Influence of Sex and Gender on the Level and Course of Subjective Cognitive Complaints After Percutaneous Coronary Intervention

**DOI:** 10.1007/s12529-024-10333-y

**Published:** 2024-11-06

**Authors:** Sophie C. M. van den Houdt, Paula M. C. Mommersteeg, Jos Widdershoven, Nina Kupper

**Affiliations:** 1 Center of Research On Psychological Disorders and Somatic Diseases (CoRPS), Department of Medical & Clinical Psychology, Tilburg University, PO Box 90153, 5000LE Tilburg, the Netherlands; 2 Department of Cardiology, Elisabeth-TweeSteden Hospital, Doctor Deelenlaan 5, 5042 AD Tilburg, the Netherlands

**Keywords:** Sex and Gender Differences, Cognitive Complaints, Coronary Heart Disease (CHD), General Linear Mixed Modeling

## Abstract

**Background:**

Cognitive complaints (e.g., health anxiety and illness disruption) are commonly experienced by patients with coronary heart disease (CHD). While it is known that sex and gender affect health, their influence on cognitive complaints remains unknown. The current study explored sex and gender differences in cognitive complaints during the first month (acute phase) and beyond, up to 2 years (recovery phase) after percutaneous coronary intervention (PCI).

**Method:**

A total of 514 (*M*_age_ = 64.2 ± 8.9, 84.2% male) completed the cognitive scale of the Health Complaints Scale (i.e., overall cognitive complaints, health worry, illness disruption) at baseline, 1, 12, and 24 months post-PCI. Additional questionnaires to gauge gender identity, traits, and norms were administered in a follow-up study. General linear mixed model analyses were conducted to examine the influence of sex, gender, sex × gender, and covariates on cognitive complaints.

**Results:**

Cognitive complaints only decreased in the first month post-PCI (all *p* < .01). There were no clear sex differences. However, gender generally predicted cognitive complaints in the first 2 years post-PCI: masculinity was related to less complaints, whereas feminine individuals reported higher absolute levels of complaints.

**Conclusion:**

The current findings reveal that gender is a valuable factor concerning health complaints and indicate the need to further elaborate on the discrepancies between sex and gender in health research.

**Supplementary Information:**

The online version contains supplementary material available at https://doi.org/10.1007/s12529-024-10333-y.

## Introduction

Cardiovascular disease (CVD) has been the leading cause of morbidity and mortality for decades [1, 2], with incidence rates rapidly increasing [3]. Even though males and females have a similar actual lifetime risk of CVD [4, 5], CVD is largely perceived as a man’s disease [6–8]. There are important sex differences that often remain unconsidered: in every age group up to 75, males have a higher risk of developing and passing from coronary heart disease (CHD) [9, 10], while older-aged females have higher absolute numbers of CVD mortality and strokes [6, 9, 10]. Fortunately, with continuous progress in treatment options, cardiac mortality has decreased in the last decades in both males and females [11] which shifted the research focus on patient-reported outcomes, such as perceived symptom reports and quality of life [12].

Among patients with established CHD, their experienced health complaints were found to reduce their quality of life [13] and increase the risk of mortality [14]. Health complaints do not necessarily only pertain to bodily symptoms but may also include psychological and behavioral aspects (e.g., health anxiety). In some instances, health anxiety may be more prominent than bodily symptoms confirming the multifaceted character of health complaints [15]. Besides a fear of death, patients with CHD often experience anxiety regarding their occupational, physical, and social functioning [16]. Furthermore, a disruption of daily life and increased levels of worry and apprehension are commonly reported [17].

While it is unknown whether male and female patients experience these cognitive health complaints in a different way, females more often perceive, evaluate, and act differently upon their symptoms [18–20]. Previous research found that females tend to report a poorer subjective health status and commonly adopt illness behavior in the form of bed rest and restricted activity [20]. Furthermore, females are more outspoken about their subjective health complaints and generally are more inclined to report the symptoms they experience [20]. Males, on the other hand, have the tendency to be concerned about their portrayed identity rather than communicating their symptoms [21]. Moreover, their capacity to recognize symptoms might be constrained by societal views, in which males are often expected to be stoic and ignore their symptoms [20].

While there is convincing evidence for the role of sex differences regarding CHD, the overall burden has stagnated in female patients [22]. Therefore, further exploring heterogeneity may lead to a better understanding how males and females differ in their burden, such as health complaints. Sex differences in health complaints are believed to largely originate from social perspectives, such as socialization, social positions, and traditional sex roles [20]. Hence, more research calls to explore the role of gender aside from differences with respect to biological sex [23, 24]. While sex and gender are often viewed as the same construct, there are considerable differences between both concepts. Sex refers to those attributes that are of biological nature such as chromosomes, reproductive organs, and hormones [25–27] and is believed to *interact* with gender [26], which is a sociocultural construct consisting of multiple dimensions that affect norms, identities, and behaviors of individuals [23, 26, 28]. Gender is traditionally classified in feminine (e.g., gentle, compassionate) and masculine characteristics (e.g., dominant, leading) [29]. However, both can range in different extents in males and females, coexist or be non-existent [30], and may have a differential expression in various contexts [26, 31]. The interaction between sex and gender may lead to diversity in risks, health outcomes, and vulnerabilities [32].

Masculinity was previously found to be related to a higher risk of developing CHD [33, 34] as well as unfavorable health behaviors [35]. On the contrary, a lower risk of CHD-related mortality was previously found in males who were more feminine [36]. This could be explained by the social expectation that femininity is related to a higher concern for health [20, 36]. Symptom reporting is likewise characterized by gender differences: gendered characteristics related to presumed womanhood (e.g., [37, 38]), such as unemployment [20], widowhood [20], and caretaking and household responsibilities [39] may explain the higher level of symptom reporting among females. Males, on the other hand, are more often socialized to ignore their symptoms and expected to be more stoic [20] and less vulnerable [40]. However, it is unclear to what extent sex, gender, and their interaction impact cognitive health symptoms while previous findings call for more in-depth exploration of cognitive health complaints among patients with heart disease.

Furthermore, it is unknown whether cognitive complaints fluctuate after percutaneous coronary intervention (PCI) for either acute coronary syndrome or chronic angina to relieve chronic chest pain. Especially in the first weeks, complaints may differ as patients yet have to receive more information and ways to cope [41]. Therefore, the first research aim examines how the level and course of cognitive health complaints fluctuates among patients after PCI, particularly in the first month (acute phase) compared to the recovery phase (1 month–2 years). It was hypothesized that cognitive complaints were more pronounced during the first month, as compared to the recovery phase. As a second research aim, the current study assessed how sex relates to the level and course of cognitive health complaints, with the hypothesis that cognitive complaints decrease for both males and females, but that females report higher scores on cognitive complaints overall based on previous research on symptom reporting and perception, as well as the behavior following illness (e.g., [18–20]). Additionally, the third research aim explored the main effects as well as the moderating role of gender on the relationship between sex and cognitive health complaints to assess whether gender may diminish or augment the effect of sex on these complaints. Feminine characteristics were hypothesized to relate to more complaints overall (e.g., [20]).

## Methods

### Study Design and Population

The current study comprises data from both a longitudinal cohort study (i.e., the main study) and a sub-study as part of the Tilburg Health Outcomes Registry of Emotional Stress after Coronary Intervention (THORESCI) study which follows participants for a period of 2 years after undergoing an acute or elective PCI treatment at the Elisabeth-TweeSteden (ETZ) hospital in Tilburg, the Netherlands. Participants were included up until 5 days after their PCI and filled out the baseline questionnaire (T0) at bedside or through the telephone in case they were released from the hospital, which was often the case for patients with an elective PCI. Follow-up questionnaires were administered at 1 (T1), 6 (T2), 12 (T3), and 24 (T4) months after PCI by mail or online through Qualtrics survey software. When approached by the research assistants at T0, patients first were provided with an information letter, informed consent form, and opportunity to ask further questions before receiving the first set of baseline questionnaires. In addition to the data collected in the main study, additional gender-related data were collected from November 2020 to January 2021 in a separate sub-study. This additional data collection included participants who had completed the main study between 2013 and 2019, as well as those who had not yet completed the study. Again, participants first were provided with more information and had to provide consent before filling out the questionnaires.

Eligibility criteria included sufficient understanding of the Dutch language (written and spoken) and being at least 18 years old. Furthermore, patients were excluded from participation in case of life-threatening comorbidities (e.g., metastasized cancer), severe cognitive disorders (e.g., dementia), or a disability that would prevent them from filling out the questionnaires (e.g., blindness). The study protocol and its amendments were approved by the institutional medical ethical review board (METC Brabant, reference number NL46259.028.13), and was in line with the Helsinki declaration.

### Measures

#### Patient Characteristics

Demographics and medical characteristics at baseline (T0) were obtained through both self-report and by the electronic patient records. The demographics included sex and age. PCI indication (acute or elective), cardiac history (yes/no; e.g., previous PCI, CABG, or myocardial infarction), and comorbidities (yes/no; e.g., cancer in the last 5 years, kidney disease, anemia, diabetes mellitus) belonged to the medical characteristics that were used as medical covariates in the main analyses.

#### Gender

##### Gender Traits

Feminine and masculine gender traits were measured by the short version of the Bem Sex-Role Inventory (BSRI) [42] which consists of six items that are viewed as typically masculine and six items that are typically feminine. A two-factor structure (i.e., masculinity and femininity) of the Dutch BSRI was confirmed in previous research [43]. Each characteristic was scored on a 7-point scale that ranged from “never to almost never” (1) to “always” (7) with a higher score on either the masculine or feminine items indicating a higher level of masculinity and femininity, respectively. To avoid possible bias caused by dichotomization of each scale, the continuous scores for both scales were kept. Both the masculinity (*α* = 0.854) and femininity (*α* = 0.900) subscale had a good level of internal consistency.

##### Gender Identity

Participants were asked whether they perceived their gender identity as feminine, masculine, or neither by adjusting the score on a continuous slider ranging from − 50 (100% masculine) via zero (neither) to 50 (100% feminine). To ensure an easier interpretation within the analysis, a recoded scale was created that ranges from 0 (masculine) to (100 feminine).

##### Gender Norm Score

To assess gender norms, a gender norm score was composed by taking five caretaking and sociodemographic variables. Primary earner status, education level, civil status, occupational status, and household task division were used based on both availability in the dataset and recommendations from previous studies [23, 27, 44]. Following the approach of Smith and Koehoorn [44], gender-sensitive variables were recoded by examining their prevalence in males and females separately. Then, weights were assigned as follows: 0 to answers most prevalent among males, 2 to answers more prevalent among females, and 1 to categories in-between. Summing these recoded items produced a gender norm score ranging from 0 (more masculine norms) to 10 (more feminine norms). Up to two missing items were allowed and replaced by the mean of the available items. A more detailed explanation of the gender norm score can be found elsewhere [45].

#### Cognitive Complaints

The Health Complaints Scale (HCS) was used to assess self-rated cognitive complaints [46, 47] for all timepoints of the current study. Though the HCS comprises two subscales of each 12 items, merely the items of the cognitive subscale as well as its cluster scales were considered to separately assess health worry and illness disruption by six items each. Examples of items measuring health worry are “being afraid of illness” and “all worries over if physically healthy,” and two sample items of illness disruption are “not being able to work fluently” and “feeling like you are not able to do much.” The items were scored on a 5-point scale ranging from “not at all” (0) to “extremely” (4) with higher scores depicting higher levels of cognitive complaints [47]. The internal consistency in this study was found to be excellent for all timepoints for the cognitive complaint subscale (≥ 0.95), as well as the clustered scales (≥ 0.92).

### Statistical Analysis

All statistical analyses were performed using IBM SPSS statistics 27.0 for Windows [48]. First, sex-stratified analyses were performed to assess sex differences in descriptive statistics by one-way ANOVAs for continuous variables and chi-squared tests for categorical variables. Furthermore, point-biserial correlations between the gender measures and sex were calculated.

General linear mixed models (GLMM) were used to answer the current study’s research questions. GLMMs are suitable to analyze repeated measurement data since it accounts for the possibility that data is correlated. Furthermore, missing values at one certain time point do not lead to exclusion from further analysis if other data are present at other time points which limits bias while maintaining statistical power.

Multiple GLMMs were performed to gain a better understanding of how sex and gender influenced cognitive complaints over time. The first GLMM examined whether cognitive complaints as well as the separate clusters of health worries and illness disruption changed over time by adding time as the only predictor (model 1). In the second model, sex was added as a predictor to measure sex differences in complaints over time. All gender variables were assessed separately and therefore each analysis was carried out three times for the overall scale of cognitive complaints, health worries, and illness disruption. The model 3-analyses included masculine and feminine gender traits, the model-4 analyses included gender norms, and the model 5-analyses included gender identity. The main effects of gender alongside time were tested first in models that we refer to as a-models (i.e., models 3a, 4a, and 5a). In the b-models, the main effects of sex and gender as well as their interaction were tested. Expanding upon these models, the c-models also included medical covariates (i.e., cardiac history, comorbidity, PCI indication, hyperlipidemia, hypertension) and age. Before the analysis, the best covariance structures were assessed, which revealed that the models with unstructured covariance matrices and fixed effects performed best based on the log-likelihood. Furthermore, the outcomes of the correlational analysis were considered, leading to the omittance of sex from models 5b and 5c as the correlation between sex and gender identity was above 0.8. Since both the acute phase (T0–T1) and the recovery phase (T1–T3–T4) were considered in the current study, all analyses were done for both phases separately. A statistical test was considered significant when the *p*-value was ≤ 0.05, but trends are also reported (*p* < 0.10) to explore potential effects.

### Power Analysis

The minimum sample size to reach a sufficient level of power was calculated by G*Power 3.1 [49]. To achieve a power of 0.80 given an alpha of 5%, repeated measures, five measurements, two groups, and an effect size of *f*^2^ = 0.1, a sample size of at least 474 was required [50].

## Results

### Sample Characteristics

To be included in the current study, participants were required to have taken part in the sub-questionnaire to assess gender characteristics, as well as having filled in at least one time point in the main study. This resulted in a sample of 514 participants at baseline (*M*_age_ = 64.2 ± 8.9; 84% male). The participant number decreased to 479 patients at T1 (1 month), 424 at T3 (12 months), and 346 at T4 (2 years). Part of the reduction in participants may be explained by the longitudinal character of the main study, and it should be noted that patients with missing data at earlier time points (i.e., T0 and T1) did not always drop out from the study. Fortunately, GLMMs are robust for this missing data. Sex-stratified descriptive statistics for the patient characteristics are presented in Table 1. The analyses revealed that females were more likely to have hypertension than males (*χ*^2^ (2, *N* = 514) = 4.66, *p* = 0.03).

**Table 1:** Sex-stratified demographic, clinical, and gender characteristics

	*N*	Females		Males		Test value	*p*-value
		*N*/*M*	%/SD	*N*/*M*	%/SD		
Demographics							
Sex	514	81	16%	433	84%		
Age	514	62.99	8.94	64.42	8.93	1.75	0.19
Clinical characteristics							
Hypertension	514	43	53%	174	40%	4.66	**0.031**
Hyperlipidemia	514	32	40%	148	34%	0.85	0.36
Cardiac history^1^	514	28	35%	189	44%	2.31	0.13
Comorbidty^2^	514	39	48%	162	37%	3.30	0.07
PCI indication (acute)	513	50	62%	280	65%	0.28	0.60
Gendered items							
Gender traits							
Masculine traits	487	26.41	7.15	29.46	6.48	13.25	** < 0.001**
Feminine traits	488	33.19	6.29	31.67	6.13	3.62	*0.06*
Gender identity							
Gender identification	449	87.39	15.01	14.22	19.33	833.00	** < 0.001**
Gender norm score							
Societal gender norm score^3^	428	4.93	1.34	2.99	1.67	74.22	** < 0.001**

^1^Previous myocardial infarction, coronary artery bypass grafting, percutaneous coronary intervention, pacemaker, heart failure, atrial fibrillation; ^2^diabetes, COPD, anemia, liver disease, kidney disease, cancer in the past 5 years, rheumatoid arthritis; ^3^an overview of the individual components of the societal gender norm score can be found elsewhere [45]

Bold = *p* < 0.05; italic = *p* < 0.10.

Masculinity, gender identity, and the gender norm score significantly differed between males and females (*p* < 0.05), with females reporting higher levels of feminine gendered characteristics whereas males demonstrated more masculine-related norms (*F*_(1,427)_ = 74.22, *p* < 0.001), identities (*F*_(1,448)_ = 833.00, *p* < 0.001), and traits (*F*_(1,486)_ = 13.25, *p* < 0.001). Feminine traits revealed a slight trend between males and females, with females scoring higher on femininity (*F*_(1,487)_ = 3.62, *p* = 0.058). Results also demonstrated that all gendered characteristics of the gender norm score were different between males and females, except for educational level. An overview of sex-stratified descriptive statistics of each gender measure is presented in Table 1.

### Generalized Linear Mixed Models

The findings of the GLMM are presented in Table 2 for the acute phase and Table 3 for the recovery phase. To enhance the clarity and emphasize the key messages pertaining to the research objectives, only models with time, sex, gender, and interaction variables were included. Since the results of the c-models remained consistent when age and medical covariates were controlled for, the adjusted values for sex, gender, and the sex × gender interaction in Table 2 (acute) and Table 3 (recovery) are displayed, while the complete models are available in *Supplemental Table 1*(acute) and *Supplemental 2* (recovery).

**Table 2:** Results from multivariable linear mixed models for cognitive health complaints during the acute phase (baseline — first month)

**Model 1**	**Cognitive complaints**			**Health worry**			**Illness disruption**		
	**Est**	***t*-value**	***p*-value**	**Est**	***t*-value**	***p*-value**	**Est**	***t*-value**	***p*-value**
Time — baseline (0 m)	*Reference*			*Reference*			*Reference*		
Time — 1 month (1 m)	** − 1.83**	** − 4.58**	** < 0.001**	** − 1.09**	** − 5.28**	** < 0.001**	** − 0.73**	** − 3.26**	**0.001**
**Model 2**									
Time — baseline (0 m)	*Reference*			*Reference*			*Reference*		
Time — 1 month (1 m)	** − 3.47**	** − 3.47**	** < 0.001**	** − 1.85**	** − 3.56**	** < 0.001**	** − 1.62**	** − 2.88**	**0.004**
Sex (female)	1.90	1.60	0.11	0.78	1.34	0.18	*1.13*	*1.66*	*0.097*
0 m × sex (f)	*Reference*			*Reference*			*Reference*		
1 m × sex (f)	* − 1.95*	*1.78*	*0.08*	− 0.89	− 1.58	0.12	* − 1.05*	* − 1.71*	*0.09*
**Model 3 — gender traits**	**Cognitive complaints**			**Health worry**			**Illness disruption**		
**Model 3a**	**Est**	***t*-value**	***p*-value**	**Est**	***t*-value**	***p*-value**	**Est**	***t*-value**	***p*-value**
Masculinity	** − 0.20**	** − 2.70**	**0.007**	** − 0.09**	** − 2.30**	**0.022**	** − 0.12**	** − 2.78**	**0.006**
Femininity	**0.16**	**1.96**	**0.050**	0.06	1.49	0.14	**0.10**	**2.18**	**0.030**
Time — baseline (0 m)	*Reference*			*Reference*			*Reference*		
Time — 1 month (1 m)	0.63	0.27	0.79	0.50	0.42	0.69	0.13	0.10	0.92
0 m × masculine traits	*Reference*			*Reference*			*Reference*		
1 m × masculine traits	− 0.01	− 0.07	0.95	0.01	0.30	0.77	− 0.02	− 0.40	0.69
0 m × feminine traits	*Reference*			*Reference*			*Reference*		
1 m × feminine traits	− 0.07	− 0.98	0.33	− 0.06	− 1.58	0.12	− 0.01	− 0.28	0.78
**Model 3c**									
Masculinity	− 0.10	− 0.56	0.40	− 0.09	− 1.04	0.30	− 0.01	− 0.09	0.93
Femininity	0.19	1.09	0.27	0.11	1.24	0.22	0.08	0.86	0.39
Sex (female)	− 1.75	− 0.29	0.97	− 0.16	− 0.06	0.96	− 1.58	− 0.47	0.64
Sex (f) × masculine traits	0.08	0.45	0.88	− 0.02	− 0.18	0.86	1.00	0.95	0.34
Sex (f) × feminine traits	0.05	0.27	0.81	0.05	0.51	0.61	0.01	0.04	0.97
**Model 4 — gender norm score**^**a**^	**Cognitive complaints**			**Health worry**			**Illness disruption**		
**Model 4a**	**Est**	***t*-value**	***p*-value**	**Est**	***t*-value**	***p*-value**	**Est**	***t*-value**	***p*-value**
Gender norms	**0.82**	**3.21**	**0.001**	**0.38**	**0.30**	**0.003**	**0.44**	**2.98**	**0.003**
Time — baseline (0 m)	*Reference*			*Reference*			*Reference*		
Time — 1 month (1 m)	− 0.57	0.71	0.48	− 0.52	− 1.25	0.21	− 0.06	− 0.13	0.90
0 m × gender norms	*Reference*			*Reference*			*Reference*		
1 m × gender norms	− 0.35	− 1.63	0.10	− 0.17	− 1.50	0.13	− 0.18	− 1.43	0.15
**Model 4b**									
Gender norms	0.13	0.15	0.88	0.29	0.69	0.49	− 0.16	− 0.33	0.74
Sex (female)	4.80	1.10	0.27	1.03	0.47	0.64	3.79	1.55	0.12
Sex (w) × gender norms	− 0.85	− 0.97	0.33	− 0.15	− 0.33	0.74	− 0.71	− 1.44	0.15
**Model 4c**									
Gender norms	0.04	0.05	0.96	0.25	0.63	0.53	− 0.21	− 0.47	0.64
Sex (female)	4.80	1.14	0.26	0.98	− 0.46	0.64	3.85	1.62	0.11
Sex (f) × gender norms	− 0.91	− 1.06	0.29	− 0.16	− 0.37	0.71	− 0.75	− 1.57	0.12
**Model 5 — gender identity**	**Cognitive complaints**			**Health worry**			**Illness disruption**		
**Model 5a**	**Est**	***t*-value**	***p*-value**	**Est**	***t*-value**	***p*-value**	**Est**	***t*-value**	***p*-value**
Gender identity	0.02	1.21	0.23	0.01	0.93	0.36	0.01	1.33	0.18
Time — baseline (0 m)	*Reference*			*Reference*			*Reference*		
Time — 1 month (1 m)	* − 0.95*	* − 1.75*	*0.08*	** − 0.68**	** − 2.44**	**0.015**	− 0.27	− 0.87	0.38
0 m × gender	*Reference*			*Reference*			*Reference*		
1 m × gender	** − 0.04**	** − 2.67**	**0.008**	** − 0.02**	** − 2.53**	**0.012**	** − 0.02**	** − 2.43**	**0.015**
**Model 5c**									
Gender identity	**0.03**	**2.30**	**0.022**	*0.01*	*1.82*	*0.07*	**0.02**	**2.49**	**0.013**

Model 3a/4a/5a, the main effect of gender and time, as well as its interaction

Model 3b/4b, the main effects of sex and gender, as well as its interaction.

Model 3c/4c, the main effects of sex and gender and its interaction, adjusted for medical covariates and age (not displayed; see supplementary material).

Model 5c, the main effect of gender identity adjusted for medical covariates and age (not displayed; see supplementary material).

Bold = *p* < .05; italic = *p* < .10.

**Table 3:** Results from multivariable linear mixed models for cognitive health complaints during the recovery phase (1–24 months)

**Model 1**	**Cognitive complaints**			**Health worry**			**Illness disruption**		
	**Est**	***t*-value**	***p*-value**	**Est**	***t*-value**	***p*-value**	**Est**	***t*-value**	***p*-value**
Time — 1 month (1 m)	*Reference*			*Reference*			*Reference*		
Time — 1 year (1y)	− 0.38	− 1.07	0.29	− 0.07	− 0.40	0.69	− 0.31	− 1.50	0.14
Time — 2 years (2y)	− 0.37	− 0.89	0.37	− 0.05	− 0.23	0.82	− 0.32	− 1.33	0.19
**Model 2**									
Time — 1 month (1 m)	*Reference*			*Reference*			*Reference*		
Time — 1 year (1y)	** − 1.75**	** − 1.99**	**0.048**	* − 0.78*	* − 1.68*	*0.09*	* − 0.98*	* − 1.91*	*0.06*
Time — 2 years (2y)	− 0.20	− 0.20	0.84	− 0.24	− 0.47	0.64	0.05	0.08	0.93
Sex (female)	1.88	1.57	0.12	0.78	1.33	0.18	1.10	1.60	0.11
1 m × sex (f)	*Reference*			*Reference*			*Reference*		
1y × sex (f)	* − 1.63*	* − 1.69*	*0.09*	* − 0.83*	* − 1.65*	*0.099*	− 0.80	− 1.42	0.16
2y × sex (f)	0.20	0.18	0.85	− 0.23	− 0.41	0.68	0.44	0.69	0.49
**Model 3 — gender traits**	**Cognitive complaints**			**Health worry**			**Illness disruption**		
**Model 3a**	**Est**	***t*-value**	***p*-value**	**Est**	***t*-value**	***p*-value**	**Est**	***t*-value**	***p*-value**
Masculine traits	** − 0.21**	** − 2.84**	**0.005**	** − 0.09**	** − 2.42**	**0.016**	** − 0.12**	** − 2.91**	**0.004**
Feminine traits	**0.18**	**2.26**	**0.024**	*0.07*	*1.74*	*0.08*	**0.11**	**2.47**	**0.014**
Time — 1 month (1 m)	*Reference*			*Reference*			*Reference*		
Time — 1 year (1y)	2.26	1.09	0.28	0.58	0.53	0.60	1.63	1.36	0.18
Time — 2 years (2y)	3.25	1.38	0.17	1.71	1.40	0.16	1.51	1.13	0.26
1 m × masculine traits	*Reference*			*Reference*			*Reference*		
1y × masculine traits	0.01	0.18	0.86	− 0.03	− 0.85	0.40	0.02	0.46	0.64
2y × masculine traits	0.05	0.74	0.46	− 0.06	− 1.57	0.12	0.01	0.18	0.86
1 m × feminine traits	*Reference*			*Reference*			*Reference*		
1y × feminine traits	0.07	1.13	0.26	0.004	0.12	0.91	** − 0.08**	** − 2.00**	**0.046**
2y × feminine traits	0.07	0.98	0.33	− 0.01	− 0.14	0.89	− 0.07	− 1.61	0.11
**Model 3b**									
Masculinity	− 0.28	− 1.58	0.12	* − 0.15*	* − 1.77*	*0.08*	− 0.11	− 1.12	0.26
Femininity	0.14	0.76	0.45	0.09	1.05	0.29	0.04	0.37	0.72
Sex (female)	3.28	0.53	0.60	0.91	0.30	0.77	2.16	0.62	0.54
Sex (f) × masculine traits	− 0.08	− 0.42	0.68	− 0.06	− 0.63	0.53	− 0.01	− 0.05	0.96
Sex (f) × feminine traits	0.01	0.03	0.97	0.03	0.34	0.73	− 0.04	− 0.31	0.76
**Model 3c**									
Masculine traits	− 0.22	− 1.26	0.21	− 0.12	− 1.44	0.15	− 0.06	− 0.86	0.39
Feminine traits	0.12	0.66	0.51	0.09	0.98	0.33	0.03	0.25	0.80
Sex (female)	1.33	0.22	0.83	− 0.15	− 0.05	0.96	1.30	0.38	0.71
Sex (f) × masculine traits	− 0.03	− 0.14	0.89	− 0.03	− 0.33	0.74	0.02	0.17	0.87
Sex (f) × feminine traits	0.01	0.06	0.96	0.04	0.37	0.71	− 0.03	− 0.30	0.77
**Model 4 — gender norm score**^**a**^	**Cognitive complaints**			**Health worry**			**Illness disruption**		
**Model 4a**	**Est**	***t*-value**	***p*-value**	**Est**	***t*-value**	***p*-value**	**Est**	***t*-value**	***p*-value**
Gender norms	**0.84**	**3.27**	**0.001**	**0.38**	**2.97**	**0.003**	**0.46**	**3.114**	**0.002**
Time — 1 month (1 m)	Reference			*Reference*			*Reference*		
Time — 1 year (1y)	0.77	0.99	0.33	0.36	0.89	0.38	0.39	0.848	0.40
Time — 2 years (2y)	0.25	0.29	0.77	0.13	0.30	0.76	0.13	0.252	0.80
1 m × gender norms	*Reference*			*Reference*			*Reference*		
1y × gender norms	* − 0.31*	− 1.45	0.15	− 0.10	− 0.93	0.35	− 0.200	− 1.60	0.11
2y × gender norms	* − 0.18*	− 0.78	0.44	− 0.06	− 0.50	0.62	− 0.121	− 0.89	0.37
**Model 4b**									
Gender norms	− 0.38	− 0.54	0.65	0.02	− 0.05	0.96	− 0.39	− 0.82	0.41
Sex (female)	6.05	1.39	0.17	− 1.89	− 0.89	0.38	*4.23*	*1.72*	*0.09*
Sex (f) × gender norms	− 1.19	− 1.35	0.18	0.39	0.90	0.37	− 0.82	− 1.65	0.10
**Model 4c**									
Gender norms	− 0.45	− 0.56	0.58	− 0.06	− 0.14	0.89	− 0.44	− 0.96	0.34
Sex (female)	5.86	1.39	0.17	1.77	0.86	0.39	*4.21*	*1.76*	*0.08*
Sex (f) × gender norms	− 1.20	− 1.40	0.16	− 0.39	− 0.92	0.36	* − 0.84*	* − 1.74*	*0.08*
**Model 5 — gender identity**	**Cognitive complaints**			**Health worry**			**Illness disruption**		
**Model 5a**	**Est**	***t*-value**	***p*-value**	**Est**	***t*-value**	***p*-value**	**Est**	***t*-value**	***p*-value**
Gender identity	0.02	1.06	0.29	0.01	0.81	0.42	0.01	1.16	0.25
Time — 1 month (1 m)	*Reference*			*Reference*			*Reference*		
Time — 1 year (1y)	0.26	0.54	0.59	0.21	0.83	0.41	0.05	0.18	0.86
Time — 2 years (2y)	− 0.04	− 0.08	0.94	0.17	0.59	0.56	− 0.22	− 0.68	0.50
1 m × gender identity	*Reference*			*Reference*			*Reference*		
1y × gender identity	− 0.02	− 1.49	0.14	− 0.01	− 1.26	0.21	− 0.01	− 1.43	0.15
2y × gender identity	− 0.02	− 1.09	0.28	− 0.01	− 1.28	0.20	− 0.01	− 0.73	0.47
**Model 5c**									
Gender identity	0.00	0.29	0.77	− 0.00	− 0.03	0.98	0.00	0.54	0.59

Model 3a/4a/5a, the main effect of gender and time, as well as its interaction

Model 3b/4b, the main effects of sex and gender, as well as its interaction.

Model 3c/4c, the main effects of sex and gender and its interaction, adjusted for medical covariates and age (not displayed; see supplementary material).

Model 5c, the main effect of gender identity adjusted for medical covariates and age (not displayed; see supplementary material).

Bold = *p* < .05; italic = *p* < .10.

#### Time

As for time (model 1), during the first month, a significant decrease was observed for the full scale of cognitive complaints (*B* =  − 1.83, *p* < 0.001; Δ − 1.85), as well as both subscales health worry (*B* = 1.09, *p* < 0.001; Δ − 1.12) and illness disruption (*B* =  − 0.73, *p* = 0.001; Δ − 0.73). However, this decrease stagnated after the first month with no changes in complaints in the 2 years after PCI (all *p* > 0.05).

#### Sex

**Fig. 1:**
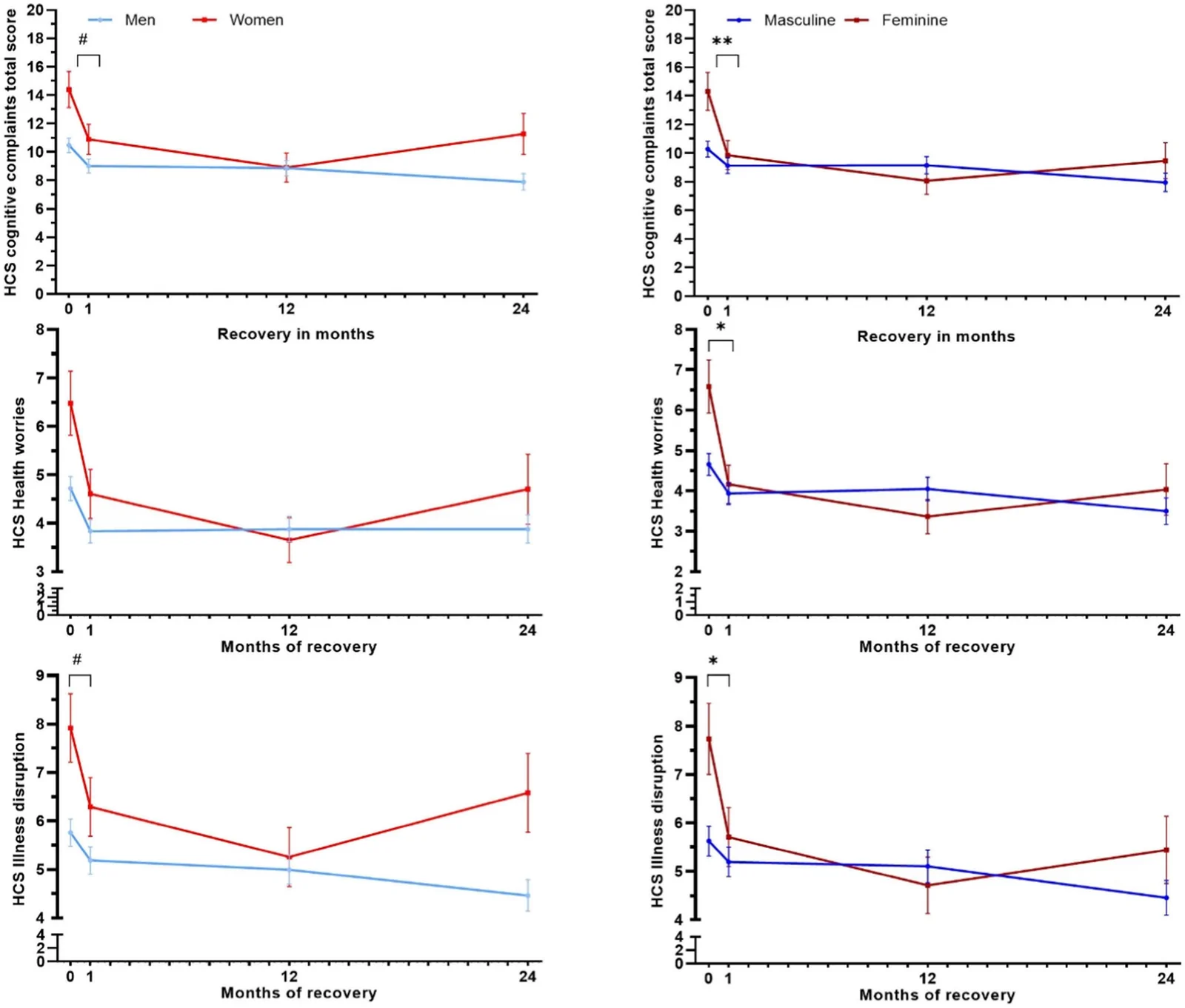
An overview of all cognitive complaints over time for males and females separately (left) and for individuals with masculine and feminine identities separately (right). The error bars represent the standard error of the mean (SEM), providing an estimate of the variability of the means within each group. Double asterisks denote interaction with time *p* < 0.01; a single asterisk denotes interaction with time *p* < 0.05; a number sign denotes interaction with time *p* < 0.10

A trending effect of sex on illness disruption indicated females experienced more illness disruptive complaints in the first month post-PCI (*B* = 1.13, *p* = 0.09; model 2), while overall cognitive complaints and health worry were the same between males and females in the 2 years after PCI. Additionally, trending interaction effects between time and sex (model 2) revealed that females, as compared to males, had a larger decline in overall cognitive complaints (*B* =  − 1.95, *p* = 0.08) and feelings of illness disruption (*B* =  − 1.05, *p* = 0.09) within the first month. In the recovery phase, trending interaction effects again demonstrated a larger decline in overall cognitive complaints (*B* =  − 1.63, *p* = 0.09) and health worry (*B* =  − 0.83, *p* = 0.099) at 1 year in females as compared to males. Figure 1 presents an overview of the cognitive complaints for males and females separately (left panel), demonstrating large differences in the first month followed by similar levels of cognitive complaints and a lower level of health worry for females, as compared to males, after 1 year post-PCI. Interestingly, while males demonstrated a decrease in overall cognitive complaints and illness disruption, as well as a stagnation in health worry in the second year, females reported increasing complaints from year 1 to year 2.

#### Gender Traits

The main effects of masculine and feminine traits (model 3a) both related to cognitive complaint experience. Masculine individuals reported lower levels of complaints throughout the course of 2 years following PCI (all *p* < 0.05). Femininity, on the other hand, was associated with higher levels of cognitive complaints and illness disruption in both phases, but not health worry. However, a trending effect implied a higher level of complaints in the recovery phase (*B* = 0.07, *p* = 0.08). All effects disappeared when controlling for the covariates (model 3b–3c).

#### Gender Norms

Patients with feminine gender norms reported more cognitive complaints, health worry, and illness disruption during both the acute and recovery phase (all *p* < 0.005; model 4a). While these effects disappeared in subsequent models, the interaction effect between sex and gender norms on illness disruption was trending toward significance in the first 2 years after PCI (*B* =  − 0.84, *p* = 0.08; model 4c). This indicated that gender non-conformity was related to more illness disruptive complaints: i.e., males with more feminine norms and females with more masculine norms tended to experience more illness disruption throughout the course of 2 years following PCI. These effects were non-existent for overall cognitive complaints and health worry.

#### Gender Identity

In the model considering time (model 5a), interaction effects between gender identity and time revealed that feminine individuals, relative to masculine individuals, reported larger declines in overall cognitive complaints (*B* = 0.04, *p* = 0.008), health worry (*B* = 0.02, *p* = 0.012), and illness disruption (*B* = 0.02, *p* = 0.015) within the first month post-PCI. This decline is also demonstrated in Fig. 1 for individuals with masculine and feminine identities separately (right panel). When controlling for demographic and medical covariates (model 5c), the main effect of gender identity was demonstrated to be important in the first month post-PCI only: a feminine gender identity was associated with more overall cognitive complaints (*B* = 0.03, *p* = 0.022) and illness disruption (*B* = 0.02, *p* = 0.013). A trending effect was revealed for health worry, likewise indicating a higher level of health worry among feminine individuals (*B* = 0.01, *p* = 0.07). These effects became non-existent in the recovery phase.

#### Covariates

Most covariates were consistently related to the experience of cognitive complaints. Overall, older patients and patients that underwent an elective PCI reported less health worry and illness disruption complaints, whereas cardiac history and presence of hypertension were associated with increased cognitive complaints. Some (trending) effects were found for comorbidity and hypercholesterolemia, indicating increased and decreased symptoms, respectively. All covariates are displayed in *Supplemental Table 1*(acute) and *Supplemental Table 2*(recovery).

## Discussion

The current study examined the influence of sex and gender on the level and course of self-reported cognitive health complaints among patients that underwent a PCI. The results confirmed the first hypothesis that cognitive complaints declined over time as demonstrated by a higher level of complaints in the first month, followed by a decrease thereafter. As for the second aim, males and females did not show significant differences in the level of cognitive complaints, thus not supporting the second hypothesis. While some complaints were observed to have a larger decrease over time for females as compared to males, these were only trending effects and should be interpreted with caution. As for gender, the results confirmed the third hypothesis and demonstrated that feminine gender characteristics were related to higher cognitive health complaints, while masculine traits predicted a lower level of complaints. All these effects disappeared after adjustment for covariates, except for gender identity in the first month. With regard to the covariates, in most analyses, it was revealed that cardiac history, acute PCI, and hypertension were all related to a higher amount of cognitive health complaints.

Unlike the second hypothesis, sex was not related to the overall experience of cognitive complaints in the current study. Van Wijk and Kolk [20] suggested that females tend to adopt illness behavior (e.g., restricted activity) more often and are more inclined to report their symptoms to others [20, 21]. Additionally, females are believed to differ in their perception, evaluation, and behavior based on their symptoms [18–20]. The current study was unable to confirm this. However, these sex differences are believed to stem from socialization processes, as well as role expectations and norms that cause females to be more attentive and active toward the symptoms they experience [18–20]. This indicates these sex disparities may be associated with gender roles rather than being primarily linked to biological characteristics [18], which corroborates the current study’s findings as the current study merely demonstrated the predictive effect of gender, not biological sex. Additionally, with the higher older mean age in the current sample, sex differences may be minimal due to a decline in sex hormones [51–53].

The results of the current study also demonstrated that masculine traits were related to a lower level of health anxiety and illness disruption, whereas feminine traits were related to more complaints. Besides gender traits, a more feminine gender norm score and gender identity were likewise associated with more cognitive health complaints. Prior research suggested that illness behavior is influenced by multiple gendered mechanisms, such as socialization in childhood, role responsibilities among females, and social acceptability of illness, symptoms, and seeking help [18]. In addition, the association between masculine characteristics and the lower report of health anxiety and illness disruption may be related to the concern about recapturing the manly identity [21]. Furthermore, masculine gender roles are associated with less expression of vulnerability [40]. Femininity, on the other hand, is linked to being more vulnerable [40] and the tendency to communicate the symptoms that are perceived [20]. Moreover, gendered characteristics and expectations generally related to womanhood (e.g., caretaking and household responsibilities [39], widowhood [20]) were likewise associated with more symptom reporting.

The results revealed a trending effect of gender non-conformity (i.e., gender expressions that differ from societal expectations) indicating males with feminine norms and females with masculine norms reported higher levels of illness disruption during the recovery phase. An explanation may be related to experiencing more distress due to not meeting the societal gender norm [36], which in turn is related to increased symptom reports [46].

It was also found that cardiac history and comorbidity were generally related to more cognitive complaints. These findings did not surprise, given the established links with illness disruption and COPD [54], and cancer survivorship [55], as well as health anxiety and diabetes [56]. As for patients with heart disease, anxiety [16], worry, and daily life disruption [17] are commonly experienced. As for age, the current study revealed that older patients reported less cognitive complaints. This aligns with previous evidence suggesting that younger patients may experience more complaints, possibly because their cardiac health has a greater impact due to their responsibilities, such as work and family [57]. Additionally, the finding that patients with an elective PCI reported more health worry likewise corroborates prior research. Elective PCI aims to treat stable angina. However, patients with chronic pain also tend to experience high levels of health anxiety [58] which explains the current findings.

### Clinical Implications

The current study reveals that gender influences reported cognitive health complaints during the acute phase and in the 2 years after PCI. Since the treatment of health complaints belongs to the primary goals of cardiac rehabilitation (CR) by enhancing perceived health [47], it may be useful to incorporate gender constructs when screening for at-risk patients in advance of CR. For example, gender norms are presented in all aspects of everyday life [59], and can easily be captured through demographic and survey data [44]. It therefore may be argued that screening for gender norms is most efficient. As for therapeutic interventions in CR, gender-sensitive treatment approaches are less common. However, the gendered background of certain behaviors could be taken into consideration. For example, typical masculine beliefs about health could be targeted by challenging these beliefs [60]. This approach may help address the potential impact of sociocultural gender norms on one’s burden. Future research could further explore the potential of adopting such a gender-sensitive approach in the context of CR.

Moreover, the observed decline in cognitive health complaints during the first month after PCI, followed by a plateau in the 2 years after, underscores the significance of identifying these complaints and offering targeted interventions. It is crucial to address these concerns post-PCI and to periodically repeat the interventions targeting any cognitive complaints. It is recommended to do so particularly after the first year following the cardiac event [61]. Further research should further explore what this could look like in the cardiac practice.

### Limitations and Strengths

Several limitations should be considered when interpreting the current study’s findings. Participants were merely able to express their gender identity in terms of masculinity, femininity, or undifferentiated which ignored the possibility of gender fluidity [62]. Additionally, it ignores how it may vary over time and contexts [26, 31]. Another limitation pertains to the underrepresentation of females in the current study. However, this is consistent with the lower prevalence of PCI in females [63] and their limited presence in PCI patient studies [64]. Furthermore, as illustrated in the figures, there is greater variability observed among females and individuals who identify as feminine. This variability could account for the absence of certain effects in the GLMM results, which differs from what can be inferred from the visual representation in the figures. Future research could improve the generalizability and precision by including other a more diverse sample in terms of sex and gender.

Another limitation relates to the use of self-reports to operationalize health complaints. Self-reported measures are known to be susceptible to recall bias and socially desirable answers [65]. Nevertheless, it is crucial to consider self-reported items as they encompass not only physical but also psychological factors, offering a more holistic view of patients’ experiences [66, 67]. While self-reported health complaints may not correlate with the severity of cardiac disease [47], they do serve as significant predictors of increased morbidity, mortality, and overall well-being [46, 68, 69], underscoring their importance. Another limitation concerns selection bias. Those who completed all questionnaires may not represent the entire spectrum of cardiac patients, as dropouts in longitudinal tend to have worse health outcomes [70]. Nevertheless, the current findings imply that more research is needed in which comparing objective health outcomes with the course of health outcomes may broaden the current perspective.

Strengths of the current study include the longitudinal and multivariable design with several covariates. Also, the use of GLMM is a great strength of the study since this analysis minimizes the effect of dropouts on the results because patients did not have to be excluded from analysis when missing one measurement occasion. The power analysis indicated that, given the sample size in the current study, a sufficient level of statistical power was reached. It is important to note, however, that this power analysis was conducted using repeated measures. The actual power is likely even higher since GLMM typically exhibits greater statistical power compared to repeated measures designs. Previous studies have mentioned the importance of gender-specific measurements in addition to sex (e.g., [27, 71]) but usually focuses on one specific measure. By exploring multiple aspects of gender and their influence on health complaints, the current study gained a better understanding of the association between gender and health. This contributes to the current evidence and may ultimately lead to more refinement in treatment. Lastly, using the approach to studying health complaints over time provided a better insight into how cognitive health complaints change after PCI and which determinants influence these changes.

In conclusion, this study has shown that cognitive health complaints decline over time in the first month only. There were no conspicuous sex differences, but trending effects indicate a larger decline in overall cognitive and illness disruptive complaints among women in the first month post-PCI and in overall cognitive complaints and health worry in the recovery phase. These effects were also found for gender identity with all cognitive complaints in the acute period. Patients with more masculine traits reported less complaints, whereas femininity was related to higher absolute levels of complaints. Furthermore, a trending interaction effect was found, suggesting that males with more feminine norms and females with more masculine norms experienced greater illness disruption during the recovery period. This underscores the need to further study how gender non-conformity may impact perceived health. The current findings show that gender is a valuable factor concerning health complaints and indicate the need to further elaborate on the discrepancies between sex and gender in health research.

## Supplementary Information

Below is the link to the electronic supplementary material.

Supplementary file1 (DOCX 57 KB)
