# Supplementary material for: The Influence of Sex and Gender on the Level and Course of Subjective Cognitive Complaints After Percutaneous Coronary Intervention
Source: Int J Behav Med. 2024 Nov 6;33(4):553–65. doi: 10.1007/s12529-024-10333-y (PMC13623999; doi:10.1007/s12529-024-10333-y)
Supplement: Supplementary file 1 [file 12529_2024_10333_MOESM1_ESM.docx]

*Supplemental Table 1. Results from multivariable linear mixed models for cognitive health complaints during the acute phase (baseline - first month)*

|  | **Cognitive complaints** | | | **Health worry** | | | **Illness disruption** | | |
| --- | --- | --- | --- | --- | --- | --- | --- | --- | --- |
| **Model 1** | Est. | *t*-value | *p*- value | Est. | *t*-value | *p*- value | Est. | *t*-value | *p*- value |
| Time – Baseline (0m) | *Reference* | | | *Reference* | | | *Reference* | | |
| Time – One month (1m) | **-1.83** | **-4.58** | **< .001** | **-1.09** | **-5.28** | **< .001** | **-.73** | **-3.26** | **.001** |
| **Model 2** |  |  |  |  |  |  |  |  |  |
| Time – Baseline (0m) | *Reference* | | | *Reference* | | | *Reference* | | |
| Time – One month (1m) | **-3.47** | **-3.47** | **< .001** | **-1.85** | **-3.56** | **< .001** | **-1.62** | **-2.88** | **.004** |
| Sex [female] | 1.90 | 1.60 | .11 | .78 | 1.34 | .18 | *1.13* | *1.66* | *.097* |
| 0m *sex [f] | *Reference* | | | *Reference* | | | *Reference* | | |
| 1m *sex [f] | *-1.95* | *-1.78* | *.08* | -.89 | -1.58 | .12 | ***-1.05*** | ***-1.71*** | ***.09*** |
| *Gender traits* | **Cognitive complaints** | | | **Health worry** | | | **Illness disruption** | | |
| **Model 3a** | Est. | *t*-value | *p*- value | Est. | *t*-value | *p*- value | Est. | *t*-value | *p*-value |
| Masculine traits | **-.20** | **-2.70** | **.007** | **-.09** | **-2.30** | **.022** | **-.12** | **-2.78** | **.006** |
| Feminine traits | **.16** | **1.96** | **.050** | .06 | 1.49 | .137 | **.10** | **2.18** | **.030** |
| Time – Baseline (0m) | *Reference* | | | *Reference* | | | *Reference* | | |
| Time – One month (1m) | .63 | .27 | .79 | .50 | .42 | .69 | .13 | .10 | .92 |
| 0m * Masculine traits | *Reference* | | | *Reference* | | | *Reference* | | |
| 1m * Masculine traits | -.01 | -.07 | .95 | .01 | .30 | .77 | -.02 | -.40 | .69 |
| 0m * Feminine traits | *Reference* | | | *Reference* | | | *Reference* | | |
| 1m * Feminine traits | -.07 | -.98 | .33 | -.06 | -1.58 | .12 | -.01 | -.28 | .78 |
| **Model 3b** |  |  |  |  |  |  |  |  |  |
| Masculine traits | -.15 | -.84 | .40 | -.11 | -1.36 | .18 | -.03 | -.33 | .74 |
| Feminine traits | .20 | 1.12 | .27 | .11 | 1.26 | .21 | .09 | .89 | .38 |
| Sex [female] | .24 | .04 | .97 | -.88 | .30 | .77 | -.61 | -.18 | .86 |
| Sex [f] * Masculine traits | .02 | .15 | .88 | -.05 | -.49 | .62 | .07 | .69 | .49 |
| Sex [f] * Feminine traits | .05 | .24 | .81 | .05 | .48 | .63 | .00 | .002 | 1.00 |
| **Model 3c** |  |  |  |  |  |  |  |  |  |
| Masculine traits | -.10 | -.56 | .40 | -.09 | -1.04 | .30 | -.01 | -.09 | .93 |
| Feminine traits | .19 | 1.09 | .27 | .11 | 1.24 | .22 | .08 | .86 | .39 |
| Sex [female] | -1.75 | -.29 | .97 | -.16 | -.06 | .96 | -1.58 | -.47 | .64 |
| Sex [f] * Masculine traits | .08 | .45 | .88 | -.02 | -.1 | .86 | 1.00 | .95 | .34 |
| Sex [f] * Feminine traits | .05 | .27 | .81 | .05 | .51 | .61 | .01 | .04 | .97 |
| Age | **-.10** | **-2.13** | **.034** | **-.05** | **-2.35** | **.019** | *-.052* | *-1.701* | *.09* |
| Cardiac history^1^ | **2.48** | **2.95** | **.003** | **1.08** | **2.61** | **.009** | **1.394** | **2.939** | **.003** |
| Comorbidity^2^ | *1.53* | *1.74* | *.08* | *.72* | *1.66* | *.098* | .82 | 1.65 | .10 |
| PCI indication [acute] | **-1.77** | **-2.07** | **.039** | **-.93** | **-2.22** | **.027** | *-.85* | *-1.76* | *.08* |
| Hypercholesterolemia | -1.07 | -1.19 | .24 | -.70 | -1.58 | .11 | -.36 | -.71 | .48 |
| Hypertension | 1.19 | 1.31 | .19 | .66 | 1.48 | .14 | .53 | 1.04 | .30 |
| ***Gender norm score^a^*** | **Cognitive complaints** | | | **Health worry** | | | **Illness disruption** | | |
| **Model 4a** | Est. | *t*-value | *p*- value | Est. | *t*-value | *p*- value | Est. | *t*-value | *p*-value |
| Gender norms | **.82** | **3.21** | **.001** | **.38** | **.30** | **.003** | **.44** | **2.98** | **.003** |
| Time – Baseline (0m) | *Reference* | | | *Reference* | | | *Reference* | | |
| Time – One month (1m) | -.57 | .71 | .48 | -.52 | -1.25 | .21 | .06 | .13 | .90 |
| 0m * Gender norms | *Reference* | | | *Reference* | | | *Reference* | | |
| 1m * Gender norms | *.35* | -1.63 | .10 | -.17 | -1.50 | .13 | .18 | 1.43 | .15 |
| **Model 4b** |  |  |  |  |  |  |  |  |  |
| Gender norms | .13 | .15 | .88 | .29 | .69 | .49 | -.16 | -.33 | .74 |
| Sex [female] | 4.80 | 1.10 | .27 | 1.03 | .47 | .64 | 3.79 | 1.55 | .12 |
| Sex [f] *Gender norms | *-.85* | *-.97* | *.33* | -.15 | -.33 | .74 | -.71 | -1.44 | .15 |
| **Model 4c** |  |  |  |  |  |  |  |  |  |
| Gender norms | .04 | .05 | .96 | .25 | .63 | .53 | -.21 | -.47 | .64 |
| Sex [female] | 4.80 | 1.14 | .26 | .98 | -.46 | .64 | 3.85 | 1.62 | .11 |
| Sex [f] *Gender norms | -.91 | -1.06 | .29 | -.16 | -.37 | .71 | -.75 | -1.57 | .12 |
| Age | **-.16** | **-3.34** | **< .001** | **-.08** | **-3.23** | **.001** | **-.08** | **-3.09** | **.002** |
| Cardiac history^1^ | **2.28** | **2.69** | **.008** | **-1.03** | **-2.42** | **.016** | **1.24** | **2.59** | **.010** |
| Comorbidity^2^ | 1.45 | 1.62 | .11 | 0.69 | 1.55 | .12 | .75 | 1.50 | .14 |
| PCI indication [acute] | **-1.90** | **-2.16** | **.031** | **-1.12** | **-2.55** | **.011** | -.79 | -1.59 | .11 |
| Hypercholesterolemia | -1.23 | -1.35 | .18 | *-0.80* | *-1.77* | *.08* | -.38 | -.73 | .46 |
| Hypertension | **2.00** | **2.22** | **.027** | **1.04** | **2.32** | **.021** | *.94* | *1.84* | *.07* |
| ***Gender identity*** | **Cognitive complaints** | | | **Health worry** | | | **Illness disruption** | | |
| **Model 5a** | Est. | *t*-value | *p*- value | Est. | *t*-value | *p*- value | Est. | *t*-value | *p*-value |
| Gender identity | .02 | 1.21 | .23 | .01 | .93 | .36 | .01 | 1.33 | .18 |
| Time – Baseline (0m) | *Reference* | | | *Reference* | | | *Reference* | | |
| Time – One month (1m) | *.95* | *1.75* | *.08* | **-.68** | **-2.44** | **.015** | .27 | .87 | .38 |
| 0m * Gender | *Reference* | | | *Reference* | | | *Reference* | | |
| 1m * Gender | **-.04** | **-2.67** | **.008** | **-.02** | **-2.53** | **.012** | **-.02** | **-2.43** | **.015** |
| **Model 5c** |  |  |  |  |  |  |  |  |  |
| Gender identity | **.03** | **2.30** | **.022** | *.01* | *1.82* | *.07* | **.02** | **2.49** | **.013** |
| Age | **-.11** | **-2.30** | **.022** | **-.06** | **-2.49** | **.013** | *-.05* | *-1.87* | *.06* |
| Cardiac history^1^ | **2.58** | **2.94** | **.003** | **-1.05** | **-2.44** | **.015** | **1.52** | **3.08** | **.002** |
| Comorbidity^2^ | *1.70* | *1.85* | *.07* | *-.78* | *-1.73* | *.084* | *.92* | *1.77* | *.08* |
| PCI indication [acute] | **-2.04** | **-2.29** | **.023** | **1.11** | **2.53** | **.012** | *-.93* | *-1.85* | *.07* |
| Hypercholesterolemia | -.77 | -.82 | .41 | .67 | 1.45 | .15 | -.09 | -.17 | .87 |
| Hypertension | 1.18 | 1.25 | .21 | -.69 | -1.49 | .14 | .49 | .92 | .36 |

**Note**. Model 3a/4a/5a: The main effect of gender and time, as well as its interaction

Model 3b/4b: The main effects of sex and gender, as well as its interaction

Model 3c/4c: The main effects of sex and gender and its interaction, adjusted for medical covariates and age (not displayed; see supplementary material)

Model 5c: The main effect of gender identity adjusted for medical covariates and age (not displayed; see supplementary material)

^1^Previous myocardial infarction, coronary artery bypass grafting, PCI, heart failure, atrial fibrillation, and implanted pacemaker ^2^Diabetes, COPD, anemia, liver disease, kidney disease, cancer in the past 5 years, rheumatoid arthritis

*Supplemental Table 2. Results from multivariable linear mixed models for cognitive health complaints during the recovery phase [1 month – 24 months]*

|  | **Cognitive complaints** | | | **Health worry** | | | **Illness disruption** | | |
| --- | --- | --- | --- | --- | --- | --- | --- | --- | --- |
| **Model 1** | Est. | *t*-value | *p*- value | Est. | *t*-value | *p*- value | Est. | *t*-value | *p*- value |
| Time – One month (1m) | *Reference* | | | *Reference* | | | *Reference* | | |
| Time – One year (1y) | -.38 | -1.07 | .29 | -.07 | -.40 | .69 | -.31 | -1.50 | .14 |
| Time – Two years (2y) | -.37 | -.89 | .37 | -.05 | -.23 | .82 | -.32 | -1.33 | .19 |
| **Model 2** |  |  |  |  |  |  |  |  |  |
| Time – One month (1m) | *Reference* | | | *Reference* | | | *Reference* | | |
| Time – One year (1y) | **-1.75** | **-1.99** | **.048** | *-.78* | *-1.68* | *.09* | *-.98* | *-1.91* | *.06* |
| Time – Two years (2y) | -.20 | -.20 | .84 | -.24 | -.47 | .64 | .05 | .08 | .93 |
| Sex [female] | 1.88 | 1.57 | .12 | .78 | 1.33 | .18 | 1.10 | 1.60 | .11 |
| 1m * sex [f] | *Reference* | | | *Reference* | | | *Reference* | | |
| 1y * sex [f] | *-1.63* | *-1.69* | *.09* | *-.83* | *-1.65* | *.099* | -.80 | -1.42 | .16 |
| 2y * sex [f] | .20 | .18 | .85 | -.23 | -.41 | .68 | .44 | .69 | .49 |
| *Gender traits* | **Cognitive complaints** | | | **Health worry** | | | **Illness disruption** | | |
| **Model 3a** | Est. | *t*-value | *p*- value | Est. | *t*-value | *p*- value | Est. | *t*-value | *p*-value |
| Masculine traits | **-.21** | **-2.84** | **.005** | **-.09** | **-2.42** | **.016** | **-.12** | **-2.91** | **.004** |
| Feminine traits | **.18** | **2.26** | **.024** | *.07* | *1.74* | *.08* | **.11** | **2.47** | **.014** |
| Time – One month (1m) | *Reference* | | | *Reference* | | | *Reference* | | |
| Time – One year (1y) | 2.26 | 1.09 | .28 | .58 | .53 | .60 | 1.63 | 1.36 | .18 |
| Time – Two years (2y) | 3.25 | 1.38 | .17 | 1.71 | 1.40 | .16 | 1.51 | 1.13 | .26 |
| 1m * Masculine traits | *Reference* | | | *Reference* | | | *Reference* | | |
| 1y * Masculine traits | .01 | .18 | .86 | -.03 | -.85 | .40 | .02 | .46 | .64 |
| 2y * Masculine traits | .05 | .74 | .46 | -.06 | -1.57 | .12 | .01 | .18 | .86 |
| 1m * Feminine traits | *Reference* | | | *Reference* | | | *Reference* | | |
| 1y * Feminine traits | .07 | 1.13 | .26 | .004 | .12 | .91 | **-.08** | **-2.00** | **.046** |
| 2y * Feminine traits | .07 | .98 | .33 | -.01 | -.14 | .89 | -.07 | -1.61 | .11 |
| **Model 3b** |  |  |  |  |  |  |  |  |  |
| Masculine traits | -.28 | -1.58 | .12 | *-.15* | *-1.77* | *.08* | -.11 | -1.12 | .26 |
| Feminine traits | .14 | .76 | .45 | .09 | 1.05 | .29 | .04 | .37 | .72 |
| Sex [female] | 3.28 | .53 | .60 | .91 | .30 | .77 | 2.16 | .62 | .54 |
| Sex [f] *Masculine traits | -.08 | -.42 | .68 | -.06 | -.63 | .53 | -.01 | -.05 | .96 |
| Sex [f] *Feminine traits | .01 | .03 | .97 | .03 | .34 | .73 | -.04 | -.31 | .76 |
| **Model 3c** |  |  |  |  |  |  |  |  |  |
| Masculine traits | -.22 | -1.26 | .21 | -.12 | -1.44 | .15 | -.06 | -.86 | .39 |
| Feminine traits | .12 | .66 | .51 | .09 | .98 | .33 | .03 | .25 | .80 |
| Sex [female] | 1.33 | .22 | .83 | -.15 | -.05 | .96 | 1.30 | .38 | .71 |
| Sex [f] *Masculine traits | -.03 | -.14 | .89 | -.03 | -.33 | .74 | .02 | .17 | .87 |
| Sex [f] *Feminine traits | .01 | .06 | .96 | .04 | .37 | .71 | -.03 | -.30 | .77 |
| Age | -.04 | -.92 | .36 | **-.02** | **-.98** | **.33** | -.02 | -.81 | .42 |
| Cardiac history^1^ | **1.93** | **2.29** | **.023** | **.90** | **2.20** | **.028** | **1.05** | **2.21** | **.028** |
| Comorbidity^2^ | **2.29** | **2.58** | **.010** | **1.06** | **2.46** | **.014** | **1.23** | **2.46** | **.014** |
| PCI indication [acute] | -1.24 | -1.43 | .15 | -.69 | -1.64 | .10 | -.49 | -1.01 | .31 |
| Hypercholesterolemia | -.80 | -.89 | .38 | -.46 | -1.06 | .29 | -.31 | -.62 | .54 |
| Hypertension | 1.38 | 1.50 | .13 | *.74* | *1.67* | *.098* | .62 | 1.20 | .23 |
| *Gender norm score^a^* | **Cognitive complaints** | | | **Health worry** | | | **Illness disruption** | | |
| **Model 4a** | Est. | *t*-value | *p*- value | Est. | *t*-value | *p*- value | Est. | *t*-value | *p*-value |
| Gender norms | **.84** | **3.27** | **.001** | **.38** | **2.97** | **.003** | **.46** | **3.114** | **.002** |
| Time – One month (1m) | Reference | | | *Reference* | | | *Reference* | | |
| Time – One year (1y) | .77 | .99 | .33 | .36 | .89 | .38 | .39 | .848 | .40 |
| Time – Two years (2y) | .25 | .29 | .77 | .13 | .30 | .76 | .13 | .252 | .80 |
| 1m * Gender norms | *Reference* | | | *Reference* | | | *Reference* | | |
| 1y * Gender norms | *-.31* | -1.45 | .15 | -.10 | -.93 | .35 | -.200 | -1.60 | .11 |
| 2y * Gender norms | *-.18* | -.78 | .44 | -.06 | -.50 | .62 | -.121 | -.89 | .37 |
| **Model 4b** |  |  |  |  |  |  |  |  |  |
| Gender norms | -.38 | -.54 | .65 | .02 | -.05 | .96 | -.39 | -.82 | .41 |
| Sex [female] | 6.05 | 1.39 | .17 | -1.89 | -.89 | .38 | *4.23* | *1.72* | *.09* |
| Sex [f] *Gender norms | -1.19 | -1.35 | .18 | .39 | .90 | .37 | -.82 | -1.65 | .10 |
| **Model 4c** |  |  |  |  |  |  |  |  |  |
| Gender norms | -.45 | -.56 | .58 | -.06 | -.14 | .89 | -.44 | -.96 | .34 |
| Sex [female] | 5.86 | 1.39 | .17 | 1.77 | .86 | .39 | *4.21* | *1.76* | *.08* |
| Sex [f] *Gender norms | -1.20 | -1.40 | .16 | -.39 | -.92 | .36 | *-.84* | *-1.74* | *.08* |
| Age | **-.14** | **-2.94** | **.003** | **-.06** | **-2.81** | **.005** | **-.07** | **-2.77** | **.006** |
| Cardiac history^1^ | **2.20** | **2.61** | **.009** | **.96** | **2.34** | **.020** | **1.25** | **2.626** | **.009** |
| Comorbidity^2^ | **1.98** | **2.23** | **.026** | **.95** | **2.19** | **.029** | **1.07** | **2.12** | **.035** |
| PCI indication [acute] | *-1.67* | *-1.91* | *.06* | **-1.00** | **-2.33** | **.021** | -.64 | -1.29 | .20 |
| Hypercholesterolemia | -1.32 | -1.47 | .14 | -.68 | -1.54 | .12 | -.61 | -1.19 | .24 |
| Hypertension | **1.95** | **2.19** | **.029** | **.99** | **2.27** | **.023** | *.90* | *-1.78* | *.08* |
| *Gender identity* | **Cognitive complaints** | | | **Health worry** | | | **Illness disruption** | | |
| **Model 5a** | Est. | *t*-value | *p*- value | Est. | *t*-value | *p*- value | **Est.** | ***t*-value** | ***p*-value** |
| Gender identity | .02 | 1.06 | .29 | .01 | .81 | .42 | .009 | 1.16 | .25 |
| Time – One month (1m) | *Reference* | | | *Reference* | | | *Reference* | | |
| Time – One year (1y) | .26 | .54 | .59 | .21 | .83 | .41 | .050 | .18 | .86 |
| Time – Two years (2y) | -.04 | -.08 | .94 | .17 | .59 | .56 | -.215 | -.68 | .50 |
| 1m * Gender identity | *Reference* | | | *Reference* | | | *Reference* | | |
| 1y * Gender identity | -.02 | -1.49 | .14 | -.01 | -1.26 | .21 | -.010 | -1.43 | .15 |
| 2y * Gender identity | -.02 | -1.09 | .28 | -.01 | -1.28 | .20 | -.010 | -.73 | .47 |
| **Model 5c** |  |  |  |  |  |  |  |  |  |
| Gender identity | .004 | .29 | .77 | -.000 | -.03 | .98 | .004 | .54 | .59 |
| Age | -.05 | -1.04 | .30 | -.03 | -1.06 | .29 | -.02 | -.93 | .35 |
| Cardiac history^1^ | **2.06** | **2.33** | **.020** | **.92** | **2.14** | **.033** | **1.15** | **2.32** | **.021** |
| Comorbidity^2^ | **2.56** | **2.75** | **.006** | **1.18** | **2.60** | **.010** | **1.39** | **2.67** | **.008** |
| PCI indication [acute] | -1.48 | -1.63 | .10 | *-.80* | *-1.82* | *.07* | -.62 | -1.21 | .23 |
| Hypercholesterolemia | -.65 | -.68 | .49 | -.45 | -.97 | .33 | -.19 | -.35 | .73 |
| Hypertension | *1.63* | *1.71* | *.09* | *.85* | *1.84* | *.07* | .74 | 1.38 | .17 |

**Note**. Model 3a/4a/5a: The main effect of gender and time, as well as its interaction

Model 3b/4b: The main effects of sex and gender, as well as its interaction

Model 3c/4c: The main effects of sex and gender and its interaction, adjusted for medical covariates and age (not displayed; see supplementary material)

Model 5c: The main effect of gender identity adjusted for medical covariates and age (not displayed; see supplementary material)

^1^Previous myocardial infarction, coronary artery bypass grafting, PCI, heart failure, atrial fibrillation, and implanted pacemaker ^2^Diabetes, COPD, anemia, liver disease, kidney disease, cancer in the past 5 years, rheumatoid arthritis
